# Managing Opioid Withdrawal in an Outpatient Setting With Lofexidine or Clonidine

**DOI:** 10.7759/cureus.27639

**Published:** 2022-08-03

**Authors:** Jeanne Gripshover, Thomas Kosten

**Affiliations:** 1 Family Medicine, Florence Medical Group, Florence, USA; 2 Epidemiology and Behavioral Science, Baylor College of Medicine, Houston, USA

**Keywords:** withdrawal, outpatients, opioid use disorder (oud), medication-assisted treatment, lofexidine, alpha-2 adrenergic agonists, abrupt discontinuation

## Abstract

Background

There is a need for improved strategies for managing abrupt opioid withdrawal when transitioning patients with opioid use disorder to comprehensive longitudinal care strategies such as naltrexone maintenance treatment. In addition, alpha-2 adrenergic agonists are used to ameliorate withdrawal symptoms, but current data characterizing real-world treatment are lacking.

Methods

A retrospective chart review was conducted in outpatients undergoing abrupt opioid withdrawal managed with lofexidine (0.18 mg, 1-4 tablets 4x daily for 7 days, pro re nata [PRN or as needed]) or clonidine (0.2 mg, 1 tablet 3x daily for 10 days, PRN). Withdrawal outcomes were characterized at 30 days of follow-up. Binomial logistic regression was used to assess a potential association of the two treatments with different likelihoods of opioid cessation success in this real-world outpatient practice.

Results

In cases treated with lofexidine (n=166) and clonidine (n=432), respectively, 40% and 10% were opioid-free, 6% and 2% continued long-term buprenorphine or methadone, 17% and 36% relapsed, and 37% and 52% were lost to follow-up at 30 days post-withdrawal. Among patients returning for follow-up care, 63% of patients treated with lofexidine and 21% treated with clonidine were opioid-free. Lofexidine was associated with a higher likelihood of opioid cessation success relative to clonidine (OR=6.47; Wald Chi-square=53.79, p<0.001).

Conclusion

Among outpatients returning for follow-up care, nearly two-thirds of those managed with lofexidine reached opioid-free status at 30 days post-withdrawal, which was a higher likelihood than those managed with clonidine, thus allowing their transition to comprehensive care, including naltrexone.

## Introduction

Fear of experiencing withdrawal symptoms is a key deterrent to opioid cessation among individuals who abuse opioids but wish to stop. Management of opioid withdrawal is a critical step in transitioning patients to long-term recovery options, such as comprehensive longitudinal care plans, including medication-assisted treatment (MAT) [1,2]. There is a growing need for non-opioid, non-addictive treatment options, considering that rapid introduction of MAT precipitates severe withdrawal [3]. Management with alpha-2 adrenergic agonists, lofexidine and clonidine, can be used to ameliorate the acute symptoms of opioid withdrawal syndrome during opioid detoxification, including anxiety, muscle pain, sweating, abdominal pain, palpitations or tachycardia, flushing and/or chills, nausea and vomiting, diarrhea, and drug cravings [4-10]. These symptoms are distressing, often debilitating, and play a key role in the high rate of early treatment failure and relapse into opioid use [3,11]. Lofexidine was approved for use in the United Kingdom in 1992 and more recently in the United States in 2018 [12]. It is the only FDA-approved non-opioid therapy indicated for acute opioid withdrawal management. Significantly more patients were reported to complete detoxification when treated with lofexidine versus placebo in FDA registration trials [12]. US practice guidelines (American Society of Addiction Medicine [ASAM]) recommend lofexidine as the preferred medication in its class for withdrawal management, especially in an outpatient setting. Whereas the UK guidelines (National Institute of Health and Care Excellence [NICE]) caution against routine clonidine treatment for opioid detoxification and instead recommend lofexidine treatment [1,2]. Clonidine is used off-label for opioid withdrawal, and clinical evidence supporting its use and symptoms-guided dosing is less robust [1,13]. Head-to-head trials have found both lofexidine and clonidine effective in managing opioid withdrawal symptoms; however, clonidine was observed to have a less favorable safety/tolerability profile due to its more potent effects on hypotension and other cardiovascular parameters [4-6,8,9]. Completion of withdrawal was also examined as a non-primary endpoint, with studies showing higher retention rates and patients feeling more in control during withdrawal treatment with lofexidine versus clonidine [4,9]. However, these head-to-head trials were conducted approximately two decades ago, primarily in Europe and with a different lofexidine dosing regimen; and present-day evidence from the US is sparse. This study characterized opioid-free status after an abrupt opioid withdrawal in outpatients, managed with lofexidine or clonidine in a real-world primary care practice.

## Materials and methods

A non-interventional, retrospective chart review was conducted for patients initiating abrupt opioid withdrawal between January 2019 and December 2020. It was managed by a nurse practitioner in family practice (Florence, Kentucky, USA), with a high volume of patients seeking opioid discontinuation. Institutional Review Board approval was obtained from Sterling IRB (ID# 9189-JGripshover); neither informed consent nor participation compensation/incentive were applicable since this was a retrospective chart review. Patients included in this analysis had presented seeking to stop abusing opioids, with a primary post-withdrawal focus in this practice being a transition to extended-release depot naltrexone maintenance. Within the study period, eligible patients who initially failed and returned for another withdrawal attempt were included in this study sample as two separate cases. All patients were managed with either lofexidine or clonidine during their abrupt opioid withdrawal. As per standard practice in this primary care center, both treatment options were discussed with patients, and treatment selection was based on their preference, health coverage, and authorization considerations. Lofexidine was prescribed as one to four 0.18 mg tablets four times daily for seven days pro re nata (PRN or as needed), consistent with the US label [12]. Clonidine was prescribed as one 0.2 mg tablet three times daily for 10 days PRN, consistent with ASAM recommendations (0.1-0.3 mg, 3-4 times daily) [1]. The therapeutic doses of clonidine most commonly used to treat hypertension have ranged between 0.2 mg and 0.6 mg daily (in divided doses), as per the US label [13].

Statistical analysis

Data were analyzed with the aim of characterizing a real-world outpatient practice. First, outcomes were examined in a descriptive analysis by categorizing all eligible cases at 30 and 180 days post-withdrawal follow-up as opioid-free, continued with the long-term opioid, relapsed (positive urine drug screen), or lost to follow-up. After excluding cases of lost to follow-up and further categorizing outcomes as cessation success or cessation failure (relapsed or continued with the long-term opioid), this study also assessed whether lofexidine and clonidine were associated with a different likelihood of opioid cessation at 30 days. Using binomial logistic regression, the unadjusted probability of being opioid-free depending on lofexidine vs. clonidine treatment was calculated, and odds ratios (ORs) and statistical significance (p≤0.05) using Wald’s Chi-squared test were determined. As a retrospective chart-based analysis of patients treated at the practice of one of the investigators (Jeanne Gripshover), power calculations were not performed, and p-values indicate nominal significance. Finally, a descriptive subgroup analysis was used to characterize study patients who previously failed on clonidine, were subsequently prescribed lofexidine and had an available follow-up of 30 days after starting either treatment. R statistical software was utilized for all descriptive and inferential analyses.

## Results

Of 598 eligible study patient cases treated with lofexidine (n=166) or clonidine (n=432) (Table 1), 74% intended to discontinue heroin or fentanyl/analogs (lofexidine, 75%; clonidine, 74%) and 93% aimed to transition post-withdrawal to extended-release naltrexone maintenance (lofexidine, 92%; clonidine, 94%). The mean age was 36.2 years (lofexidine, 35.3 years; clonidine, 36.6 years), and 47% were female (lofexidine, 46%; clonidine, 48%).

**Table 1:** Characteristics and outcomes of patient cases* initiating abrupt opioid withdrawal and managed with lofexidine or clonidine. Percentages of total cases in their respective treatment group are provided. *Of the total 598 patient cases included in the study sample, 109 patients initially failed withdrawal, then returned for another withdrawal attempt within the study period and were therefore included as 2 separate cases. ^†^Requested assisted abstinence without medication assisted maintenance (n=8) or maintenance with buprenorphine after discontinuing short-acting opioid (n=9).

	Lofexidine (N=166)	Clonidine (N=432)
Characteristics		
Age, years		
Mean (SD)	35.3 (8.8)	36.6 (9.6)
Median (range)	33.0 (19-64)	34.0 (19-68)
Sex, n (%)		
Female	76 (46)	208 (48)
Male	90 (54)	224 (52)
Opioid of abuse, n (%)		
Fentanyl/analogs	31 (19)	51 (12)
Heroin	93 (56)	267 (62)
Methadone	17 (10)	22 (5)
Prescription opioid	24 (14)	54 (13)
Missing	1 (0.6)	38 (9)
Prior lofexidine treatment, n (%)		
Yes	1 (0.6)	6 (1)
No	165 (99)	426 (99)
Prior clonidine treatment, n (%)		
Yes	165 (99)	295 (68)
No	1 (0.6)	127 (29)
Missing	0	10 (2)
Post-withdrawal treatment goal, n (%)		
Naltrexone	152 (92)	406 (94)
Other^†^	14 (8)	3 (0.7)
Missing	0	23 (5)
Post-withdrawal outcomes		
Treatment goal reached, n (%)		
Yes	92 (55)	90 (21)
No	74 (45)	342 (79)
Outcome at day 30, n (%)		
Opioid free	66 (40)	44 (10)
Relapsed	28 (17)	155 (36)
Continued long-term opioid	10 (6)	9 (2)
Lost to follow-up	62 (37)	224 (52)
Outcome at day 180, n (%)		
Opioid free	35 (21)	47 (11)
Relapsed	25 (15)	89 (21)
Continued long-term opioid	6 (4)	7 (2)
Lost to follow-up	100 (60)	289 (67)

**Figure 1:**
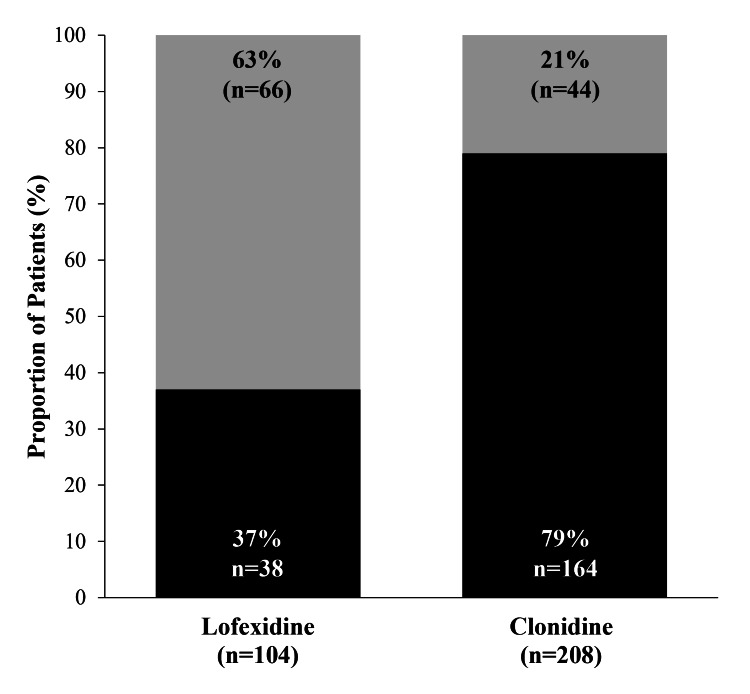
Rates of opioid cessation success (opioid-free status; gray bars) versus cessation failure (continued long-term opioid or relapsed; black bars) at 30 days after initiation of opioid withdrawal among patient cases returning for follow-up care.

Among all eligible lofexidine (n=166) or clonidine (n=432) cases, withdrawal was successfully completed, followed by a transition to maintenance naltrexone in 30% of cases (55% with lofexidine vs. 21% with clonidine). At 30 days of post-withdrawal follow-up, in all cases treated with lofexidine and clonidine, respectively, 40% vs. 10% were opioid-free, 6% vs. 2% continued long-term opioid, 17% vs. 36% relapsed, and 37% vs. 52% were lost to follow-up (Table 1). Among patients who returned for follow-up care at 30 days of follow-up, 63% of patients treated with lofexidine (n=104) and 21% treated with clonidine (n=208) were opioid-free (Figure 1), and lofexidine was associated with a higher likelihood of cessation success (i.e., opioid-free status) compared with clonidine (OR, 6.47; Wald's Chi-squared: 53.79, p<0.001). Similar trends were observed at 180 days (53% versus 33%; OR: 2.31; Wald's Chi-squared: 7.61, p=0.006). The subgroup analysis included 47 patients (mean duration of opioid abuse: 9 years) who previously failed on clonidine within 30 days after starting treatment, and subsequently received lofexidine. Of these, 27 patients (57%; mean duration of opioid abuse: 10 years) were transitioned to naltrexone within 30 days after starting lofexidine.

## Discussion

This retrospective analysis of real-world patient data demonstrated that among outpatients returning for follow-up care, those receiving lofexidine were nearly 6.5-times more likely to achieve opioid cessation success at one month than clonidine. Of those treated with lofexidine versus clonidine, respectively, 63% vs. 21% were opioid-free at one month, and better success rates were also observed with lofexidine at six months. These results show that lofexidine was more effective at mitigating withdrawal symptoms and supporting patients' transition to long-term recovery with continued management, including extended-release naltrexone in this practice. Our findings are consistent with those of randomized clinical trials comparing lofexidine and clonidine [4-6,8,9]. In those studies, lofexidine was associated with a higher percentage of successful detoxifications and better patient-reported scores for withdrawal symptoms [5], mood disturbance [5], "feeling in control" [4], and lower rates of inpatient self-discharge [9] and lower frequency of extra home nurse visits for outpatients [4]. Clinical trials have consistently demonstrated a more favorable tolerability/safety profile with lofexidine than clonidine and, specifically, fewer hypotensive symptoms with lofexidine [4-6,8,9]. Although adverse events were not collected in this retrospective analysis, our results suggest that lofexidine may have been better tolerated in this study population, with fewer outpatients lost to follow-up/abandoning treatment with lofexidine versus clonidine. The clonidine dosing used in this study was consistent with ASAM guidance [1], which recommends clonidine doses up to 3 times the FDA-approved maintenance dose for hypertension [13], which could lead to increased risk of significant cardiovascular side effects such as hypotensive symptoms. The favorable efficacy and tolerability of lofexidine relative to clonidine in this opioid withdrawal setting may be attributed partly to its unique receptor binding profile, which is similar to some newer antipsychotics [14,15]. At the receptor level, both clonidine and lofexidine bind to several subtypes of alpha-adrenoceptors (i.e., alpha-1A, alpha-2A, and alpha-2C), but lofexidine alone has demonstrated additional binding and agonist effects on serotonergic and dopaminergic receptors, including 5-HT1A, 5-HT1B, and dopamine D2S [14]. The agonist effects of lofexidine on the 5-HT1A serotonin receptor are particularly notable, as these receptors have been linked to opioid withdrawal. Serotonin levels decrease during opioid withdrawal, and 5-HT1A receptor activity regulates serotonin synthesis and release [16]. Lofexidine binding to these receptors may help maintain serotonin homeostasis during withdrawal, ameliorating withdrawal symptoms. Furthermore, lofexidine binds more strongly than clonidine to the main serum transport proteins, alpha1-acid glycoprotein (AGP) and human serum albumin (HAS) [17], resulting in less circulating free drug available to act on receptors in the body as compared to CNS, and consequently, potentially less off-target effects [17]. These pharmacologic differences may partly explain the clinical efficacy and safety differences observed between lofexidine and clonidine.

Together, the accumulated evidence suggests that in addition to improving the likelihood of successful achievement and maintenance of opioid cessation, lofexidine use may also result in less healthcare resource utilization, outside of direct medication costs, by reducing the risk of relapse, facilitating the transition to post-withdrawal maintenance, and reducing the burden on home healthcare providers. Future health-economic evaluation is therefore warranted. This study was conducted at a single site, which may limit generalizability to other settings. Considering its retrospective chart review design in a real-world setting, the study also did not control for patient characteristics. Although both treatment options were presented to patients, their treatment selection may have been influenced by healthcare coverage and authorization considerations. Thus, some patients may have been more likely to select clonidine treatment. Also, as is common in retrospective chart reviews, reasons for loss to follow-up were not documented and may have been associated with a variety of factors, including potential relapse.

## Conclusions

Compared with clonidine, management with lofexidine resulted in higher likelihood of patients remaining opioid-free at 30 days after abrupt opioid withdrawal. These findings from a real-world outpatient setting are consistent with those of randomized clinical trials and further support the guidance from both ASAM and NICE guidelines recommending lofexidine to be used over clonidine. In addition, recently published pharmacology data also provide possible pathways that may explain some of the clinical differences observed between these two medications. Taken together, these results suggest that lofexidine is an important treatment option for managing opioid withdrawal symptoms and helping outpatients transition to long-term management.
